# How We Squander Health

**Published:** 1875-01

**Authors:** 


How we Squander Health.—More human beings are killed every year by unnecessry and preventable disease than have ever been killed in any one year by war, the world over. The great majority of the human beings thus slaughtered are children. The diseases which carry them off are for the most part such as ought to be specially under the control of the women who love them, pet them, educate them, and would, in many cases, if need be, lay down their lives for them. A vast amount of disease is engendered in the sleeping-room from simple ignorance of the laws of ventilation, and in the school-room likewise, from simple ignorance of the laws of physiology. Too often from ignorance of signs of approaching disease, a child is punished for what is called idleness, listlessness, wilfulness, sulkiness; and punished, too, in the unwisest way—by an increase of tasks and confinement to the house, thus overtasking still more a brain already overtasked, and depressing still more, by robbing it of oxygen and of exercise, a system already depressed.

A medical man, passing by a school-room, heard one of his own little girls screaming and crying, and went in. The preceptress, an excellent woman, but wholly ignorant of the laws of physiology, complained that the child had of late become obstinate, and would not learn; and that, therefore, she must punish her by keeping her in doors over the unlearned lessons. The father, who knew that the child was usually a very good one, looked at her carefully for a little while; sent her out of the school-room; and then remarked that the child must not open a book for a month. If he had not acted so, he afterwards said, the child would have been dead with brain disease within the year.

Now, in the face of such facts as these, is it too much to ask of mothers, sisters, aunts, nurses, governesses—all who may be occupied in the care of children, especially of girls—that they should study somewhat the laws of life and health? Oh for a little knowledge of the laws to the neglect of which is owing so much fearful disease, which, if it does not produce immediate death, too often leaves the constitution impaired for years to come! Ah, the waste of health and strength in the young; the waste, too, of anxiety and misery in those who love and tend them! How much of it might be saved by a little rational education in those laws of Nature which are the will of God about the welfare of our bodies, and which, therefore, we are as much bound to know and obey as the spiritual laws whereon depends the welfare of our souls!

---

Growing in Favor.—The Trades Savings Bank, near the Grand Opera House, of which Mr. A. M. Lesley is President, is in a flourishing condition. The deposits are rapidly increasing, and all depositors receive 7 per cent. interest. All commissions and bonuses on loans go to the profit of depositors, and the officers get no salary. If our old readers will each secure us an additional subscriber, we may be able to deposit a little money in some sound bank before the year closes, and we know of no better one than the "Trades."

---

Bathe only in a warm room in cold weather.

